# Genetic analysis of potential biomarkers and therapeutic targets in neuroinflammation from sporadic Creutzfeldt–Jakob disease

**DOI:** 10.1038/s41598-023-41066-9

**Published:** 2023-08-29

**Authors:** Yajing Cheng, Ting Chen, Jun Hu

**Affiliations:** 1 Department of Neurology, Peking University Shenzhen Hospital, Shenzhen, China; 2 Department of Neurology, Shenzhen Second People’s Hospital, Shenzhen, China

**Keywords:** Computational biology and bioinformatics, Neurology

## Abstract

This study aimed to identify hub genes and pathological mechanisms related to neuroinflammation in Sporadic Creutzfeldt–Jakob disease (SCJD) based on comprehensive bioinformatics. SCJD and normal samples were collected from GSE160208. Weighted gene co-expression network analysis (WGCNA) and Limma R package were used to obtain key genes, which were used for enrichment and immune cell infiltration analyses. Protein–protein interaction (PPI) network, cytoHubba, and machine learning were used to screen the central genes of SCJD. The chemicals related to hub genes were predicted and explored by molecular docking. 88 candidate genes were screened. Enrichment analysis showed they were mainly related to bacterial and viral infection and immune cell activation. Immune cell infiltration analysis suggested that immune cell activation and altered activity of the immune system are involved in the progression of SCJD. After identifying hub genes, KIT and SPP1 had higher diagnostic efficacy for SCJD (AUC > 0.9), so they were identified as central genes. The molecular docking results showed hub genes both docked well with Tretinoin. KIT, SPP1, and Tretinoin are essential in developing neuroinflammation in SCJD and may provide new ideas for diagnosing and treating SCJD.

## Introduction

Creutzfeldt–Jakob disease (CJD) is a progressive and fatal neurodegenerative disease caused by misfolded, transmissible protein particles or prion proteins (PrP) that deposit in tissue to form plaques, resulting in nerve cell death and astrocyte proliferation to form spongiform encephalopathy^1^. There is a small amount of Prion protein (PrPc) in normal brain tissue, but its function is unclear. Human prion diseases are classified into sporadic (SCJD), genetic (GCJD), iatrogenic (ICJD), and new variants (VCJD). SCJD is the most common human prion disease, accounting for about 90% of cases, with an incidence rate of approximately 1.5–2.0 per million people annually. Therefore, attention should be paid to the incidence of SCJD in the population^2^. The transmission route of SCJD is currently unclear. As with many studies on animal models of prion diseases, neuroinflammation is an integral part of the pathogenesis of neurodegenerative diseases^3^. Studies on prion disease models have shown that genes expressed by activated microglia play a significant role in inflammation, metabolism, respiratory stress, and other functional regulation^4^. Therefore, exploring the mechanism of neuroinflammation in SCJD is of great significance.

With the accumulation of clinical cases and the further study of genetics and pathology, according to the polymorphism of codon 129 of the PRNP gene (Methionine/Methionine, Met/Met; Methionine/Valine, Met/Val; Valine/Valine, Val/Val) and the physical and chemical properties of PrPSc (type 1 or type 2), SCJD can be divided into 6 types: MM1, MM2, MV1, MV2, VV1, and VV2^5^. Because MM1 and MV1 are indistinguishable in clinical and pathological phenotypes, they are often combined into MM (V) 1. According to the histopathological characteristics of cortex and thalamus, MM2 can be divided into MM2 cortical type (MM2-C) and MM2 thalamic type (MM2-T, sporadic familial fatal insomnia). MV2 includes a subtype of MV2-K, also known as the kuru plaque variant. In addition, another type is defined as p-MM1 because of the widespread PrP- amyloid plaques in the white matter, which is similar to MM1 in most features^6^.

This study aims to screen key biological processes and hub genes related to neuroinflammation in SCJD and explore the pathological mechanism of neuroinflammation in SCJD based on bioinformatics analysis, machine learning, and molecular docking. We downloaded gene expression profiles related to SCJD, identified important modules related to neuroinflammation in SCJD and differentially expressed genes, and conducted clustering analysis, functional enrichment analysis, and immune infiltration analysis to explore the biological pathways related to neuroinflammation during the occurrence of SCJD. In addition, we constructed the PPI network of shared genes and used two machine-learning methods to screen critical genes related to neuroinflammation in SCJD. Then, we identified the hub genes for SCJD by taking the intersection of the top 20 genes ranked by cytoHubba, SVM-RFE, and RF. We also identified the hub genes by analyzing the ROC curve of hub genes and using another dataset related to SCJD. Finally, we explored the chemical substances related to key genes using CTD and molecular docking to explore the interaction between hub genes and chemical substances. In conclusion, bioinformatics methods were used to screen candidate genes related to neuroinflammation in SCJD, identify key genes, and explore the role of neuroinflammation in developing SCJD, providing new ideas for the diagnosis and treatment of this disease.

## Materials and methods

**Figure 1:**
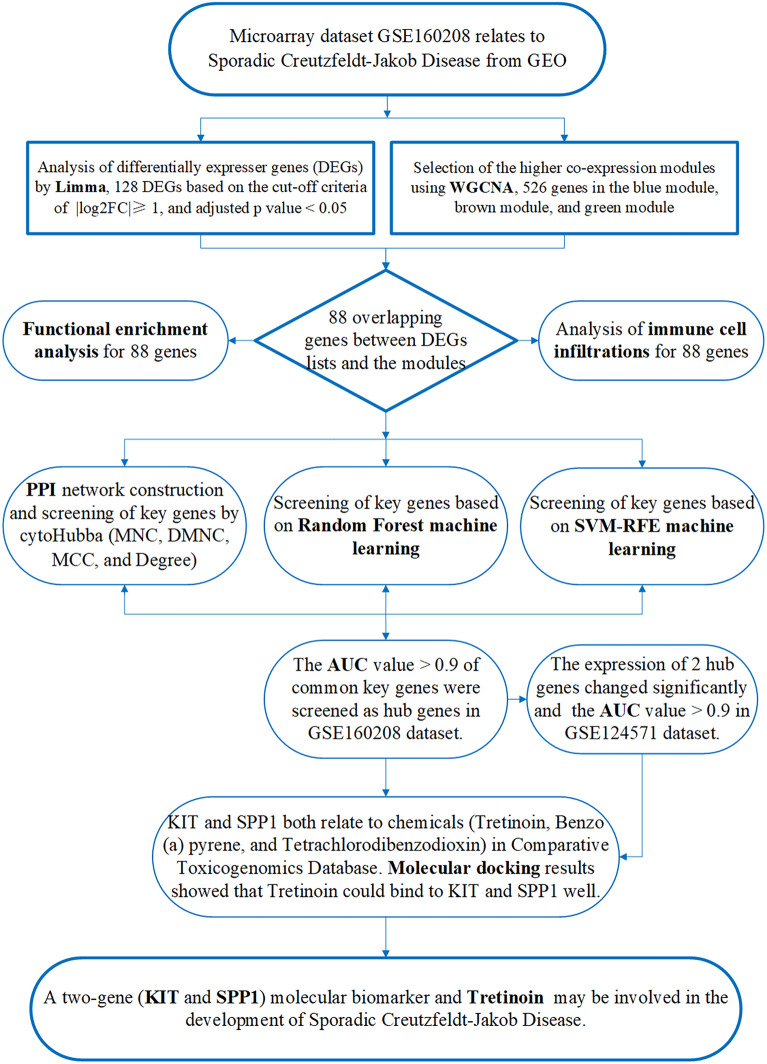
This flowchart shows the steps of bioinformatics analysis, machine learning, and molecular docking in this study.

The flowchart in Fig. 1 presents a multistep integrated analysis of this study.

### Data collection and data preprocessing

The “Sporadic Creutzfeldt–Jakob Disease” and “Neuroinflammation” were searched in the GEO (Gene Expression Omnibus) database, and the GSE160208 dataset related to neuroinflammation in SCJD was finally selected for further analysis^7^. It consists of the expression data of 800 neuroinflammation-related genes in the brain tissues of 27 SCJD patients and 20 normal individuals. The dataset also includes grouping for each sample (Control and SCJD) (Table 1), brain tissue partition (frontal cortex and cerebellum), gender (Female and Male), codon-129 mutation (MM, MV, and VV), and SCJD subtype (MM1, MM2, MV1, MV2, VV1, and VV2).

**Table 1:** Description of samples in GSE160208 used in this study.

Group	Brain. region	Gender	codon-129 mutation	SCJD subtype
27 SCJD	14 FC^①^ 13 CB^②^	13 Female 14 Male	14 MM 6 MV 7 VV	12 MM1 2 MM1 + 2 4 MV1 2 MV2 7 VV2
20 Normal	10 FC 10 CB	10 Female 10 Male	8 MM 8 MV 4 VV	–

①FC: frontal cortex;

②CB: cerebellum.

### Gene co-expression network construction and identification of key modules

The WGCNA R package was used to construct a co-expression network, and the soft threshold was estimated using the pickSoft Threshold function. Then, the correlation matrix was created based on the soft threshold, and the topological overlap matrix (TOM) was further calculated. Finally, hierarchical clustering was performed using the hclust function, and genes were grouped based on the topological overlap dissimilarity (1-TOM). The dynamic tree cut algorithm was used to determine gene modules, with a minimum module size of 30 genes and a minimum height for merging modules of 0.25 to make the module more powerful. The different modules identified by WGCNA were represented by different colors displayed on each network. The correlation between gene modules and clinical phenotype was used as a condition for screening disease-causing module genes.

### Identification of DEGs

The “limma” package was used for screening DEGs in the brain tissues of patients with sporadic Creutzfeldt–Jakob disease (SCJD) and normal individuals. The screening criteria were: | log2FC |> 1 and adj.P < 0.05. The DEGs were visualized using the “ggplot2” package of the R language. If the log2FC corresponding to the DEG is > 0, the expression of the gene is elevated in the brain tissue of patients with SCJD; if the log2FC complementary to the DEG is < 0, the DEGs are expressed lower in the brain tissue of patients with SCJD.

The “Venn Diagram” package drew a Venn diagram of the DEGs between crucial modules and SCJD. The “Complex Heatmap” package in R was used to visualize the expression levels of shared genes between the key modules and DEGs in SCJD.

### Functional enrichment analysis

Functional enrichment analysis can assign hundreds or thousands of genes to different pathways, thus reducing the complexity of the study. Using the “cluster Profiler” and “DOSE” packages in R language, KEGG pathway analysis and GO enrichment analysis was performed on candidate genes for SCJD. GO enrichment analysis covers three aspects of biology: biological processes, cellular components, and molecular functions. In this study, adj.*P* < 0.05 was used as the selection criteria, and the top ten ranked pathways were selected to explore the biological processes related to neuroinflammation in SCJD.

### Analysis of immune cell infiltrations

The CIBERSORT algorithm is widely used to evaluate the relative content and dynamic regulation process of 22 immune cells. It is superior to other methods in identifying human-resistant cell phenotypes in noise and unknown mixtures. The experiment is based on the R language program and linked to the CIBERSORT deconvolution method to calculate the distribution of 22 immune cells in the sample, including T cells, naive and memory B cells, resting and activated NK cells, etc. The calculation number is set to 1000 times, and the immune cell proportion score of each sample in the dataset is obtained. The sample’s immune cell distribution is displayed through the stacked bar chart. At the same time, the Pearson correlation coefficient is used to calculate the correlation between various immune cells and shown by bubble chart. Finally, the rank-sum test is used to analyze the expression of immune cells in the SCJD group and control group.

### Construction of PPI and screening of key genes

The selected candidate genes were imported into the STRING database (http://string.embl.de/) to construct a protein–protein interaction (PPI) network, which was further analyzed and visualized using Cytoscape software, the cytoHubba plugin. Based on four scoring algorithms, including Degree, MNC (maximum neighborhood component), MCC (maximal clique centrality), and DMNC (density of maximum neighborhood component), each gene in the PPI network was scored^8,9^. The top 20 genes were selected in each algorithm, and shared genes of the four scoring algorithms were identified as key genes. Then Pearson correlation coefficients were calculated to evaluate the correlation between key genes.

### Screening key genes of SCJD by machine learning

Machine learning algorithms can obtain more precise models and have been widely used in exploring biomarkers^10^. Support vector machine-recursive feature elimination (SVM-RFE) is a supervised machine-learning technique that sorts features recursively. Random forests (RF) generate highly accurate classification features by iteratively scoring categorical variables using a decision tree classifier model and jointly screening core genes in chip datasets. In this study, SVM-RFE is executed through the “e1071” package with fivefold cross-validation in R, and we use the SVM-RFE algorithm to screen the top 20 genes. RF is an integrated learning method based on constructing many classification trees and executed using the “randomForest” package in R to screen out the top 20 genes with importance scores. This study uses SVM-RFE and RF machine learning algorithms to analyze further the Top 20 genes screened from the candidate genes of SCJD.

### Identification of hub genes

This article uses cytoHubba, SVM-RFE, and RF algorithms to screen out each algorithm’s top 20 genes. The hub genes of SCJD were the intersection of the top 20 critical genes in three algorithms.

### Receiver operating characteristic curve analysis of hub genes

Using R language packages “pROC” and “ggplot2”, we analyzed and visualized the ROC curve of candidate hub genes and compared their diagnostic performance in SCJD. The area under the ROC curve (AUC) ranges from 0.5 to 1. The closer AUC is to 1, the better the diagnostic effect. AUC has low accuracy at 0.5–0.7, moderate precision at 0.7–0.9, and high accuracy above 0.9. The critical gene selection criteria in this study are AUC > 0.9. Finally, we identified hub genes related to SCJD neuroinflammation based on the AUC of candidate hub genes.

## Gene expression values of the hub genes in the GSE124571 dataset

The expression of hub genes in human brain tissue at the RNA level was validated using the GSE124571 dataset related to SCJD^11^, which contains ten samples of SCJD and ten samples of control brain tissue. The expression of candidate hub genes was analyzed and visualized using t-tests and the “ggplot2” package in R language. At the same time, the diagnostic efficacy of hub genes in SCJD was evaluated in the GSE124571 dataset using ROC (AUC > 0.9).

### Exploring the functions of hub genes and molecular docking analysis

The Comparative Toxicogenomics Database (CTD), released in 2004, has become a critical toxicological information resource. The CTD database describes the relationships between chemicals and genes/proteins, chemicals and diseases, genes and diseases, phenotypes, organisms, and exposure data. Through monthly updates to the CTD database, it is possible to discover new or comprehensive exposure characteristics and molecular mechanisms of chemicals, which can help form testable hypotheses on how exposure affects human health^12,13^. To explore the biological functions of hub genes, chemicals related to hub genes were predicted in CTD, and molecular docking analysis was used to provide new ideas for treating SCJD by studying the relationship between hub genes and chemicals^14^.

The protein structures of the hub genes were obtained from the Protein Data Bank (PDB) (https://www.rcsb.org/), and water molecules and heteroatoms were removed by PyMOL (V2.5.4) software. Moreover, the 3D chemical structures of the chemicals were downloaded in SDF format from the PubChem database (https://pubchem.ncbi.nlm.nih.gov/) and converted to “pdb” format by Chem3D and AutoDockTools software. Next, the proteins and chemicals were converted to “pdbqt” format files by AutoDockTools (V1.5.7). The grid box feature of AutoDockTools was used to define specific docking pockets in the selected proteins to which chemicals could bind. Once all data were prepared, the command prompt was used to perform molecular docking analysis^15,16^ and visualize the docking results with PyMOL.

### Statistical analyses

WGCNA (version 1.69), Limma (version 1.9.6), ggplot2(V3.3.3), ClusterProfiler (V3.16.0), Proc (V1.18.0), e1071, randomForest (V4.7–1.1), DOSE (V3.14.0), and GO plot (V1.0.2) were running in R (version 4.0.2) with the default statistics parameter and cut-off values specified in each section. Cytoscape (V3.8.2), PyMOL (V2.5.4), Chem3D, and AutoDockTools (V1.5.7) were also used in this study. *p < 0.05, **p < 0.01, ***p < 0.001 were defined as statistically significant.

## Results

### Weighted co-expression network construction and key module identification

**Figure 2:**
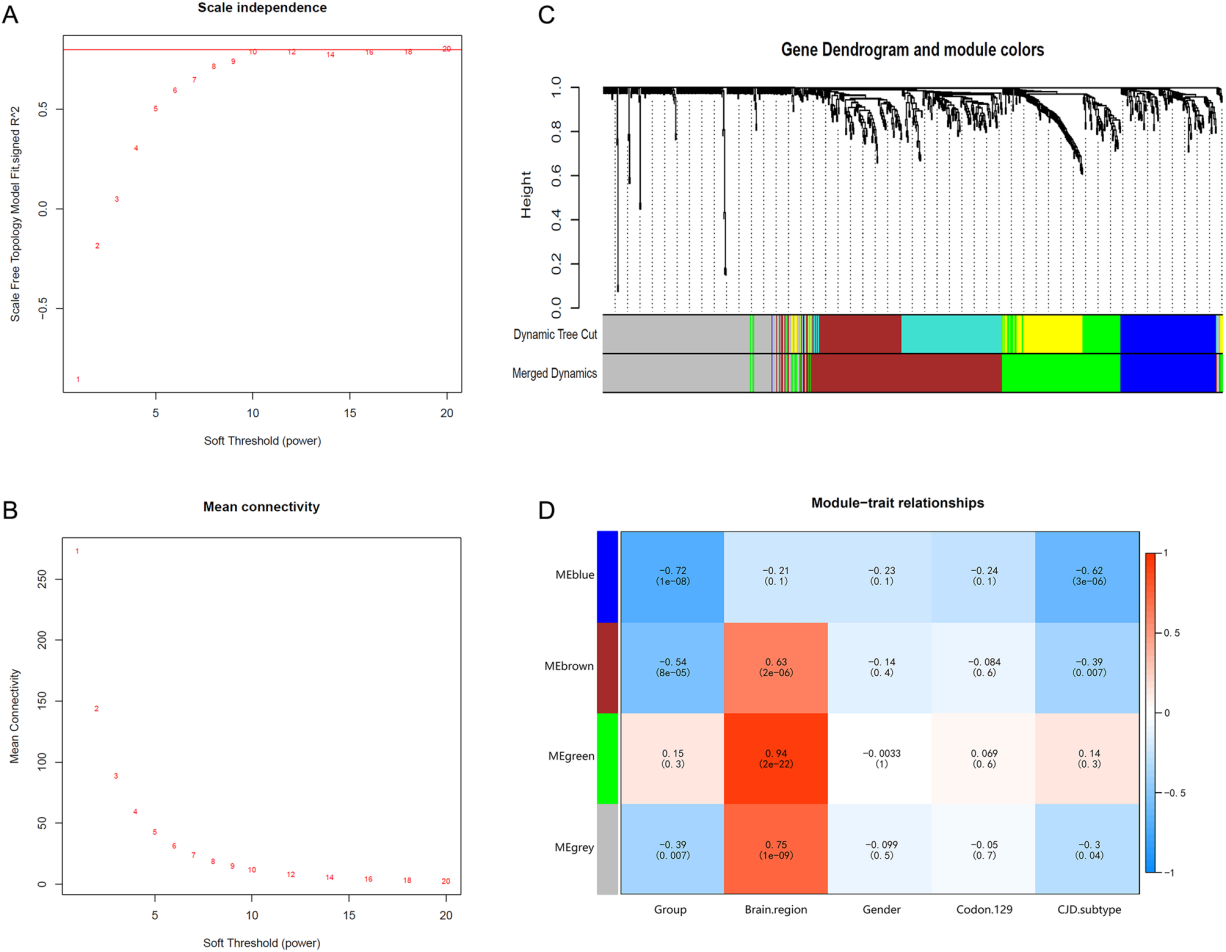
Identification of important gene modules associated with neuroinflammation in SCJD with WGCNA. (**A**) Analysis of the scale-free fit index for different soft-thresholding power. (**B**) Analysis of the mean connectivity for different soft-thresholding power. (**C**) Dendrogram of all differentially expressed genes clustered. (**D**) Heatmap of the correlation between gene modules and clinical information of SCJD.

Analysis of 800 neuroinflammation-related genes between Control and SCJD groups based on the WGCNA method. The soft threshold β value for constructing an unweighted network was chosen as 20 under the R^2^ = 0.88, calculated by pickSoftThreshold (Fig. 2A–B). The module was clustered and divided according to gene expression characteristics, and four modules (blue, brown, green, and grey) were identified (Fig. 2C–D). The relationship between modules and clinical phenotypes was calculated using characteristic vector values of each module, where blue and brown modules showed higher relevance with groupings (cor > 0.5, *P* < 0.01), and the brown, green, and grey modules were highly correlated with brain regions (cor > 0.5, *P* < 0.01) (Fig. 2D). In addition, because the genes in the grey module could not be clustered, we selected the 526 genes included in the blue, brown, and green modules for further analysis.

### Identification of DEGs

**Figure 3:**
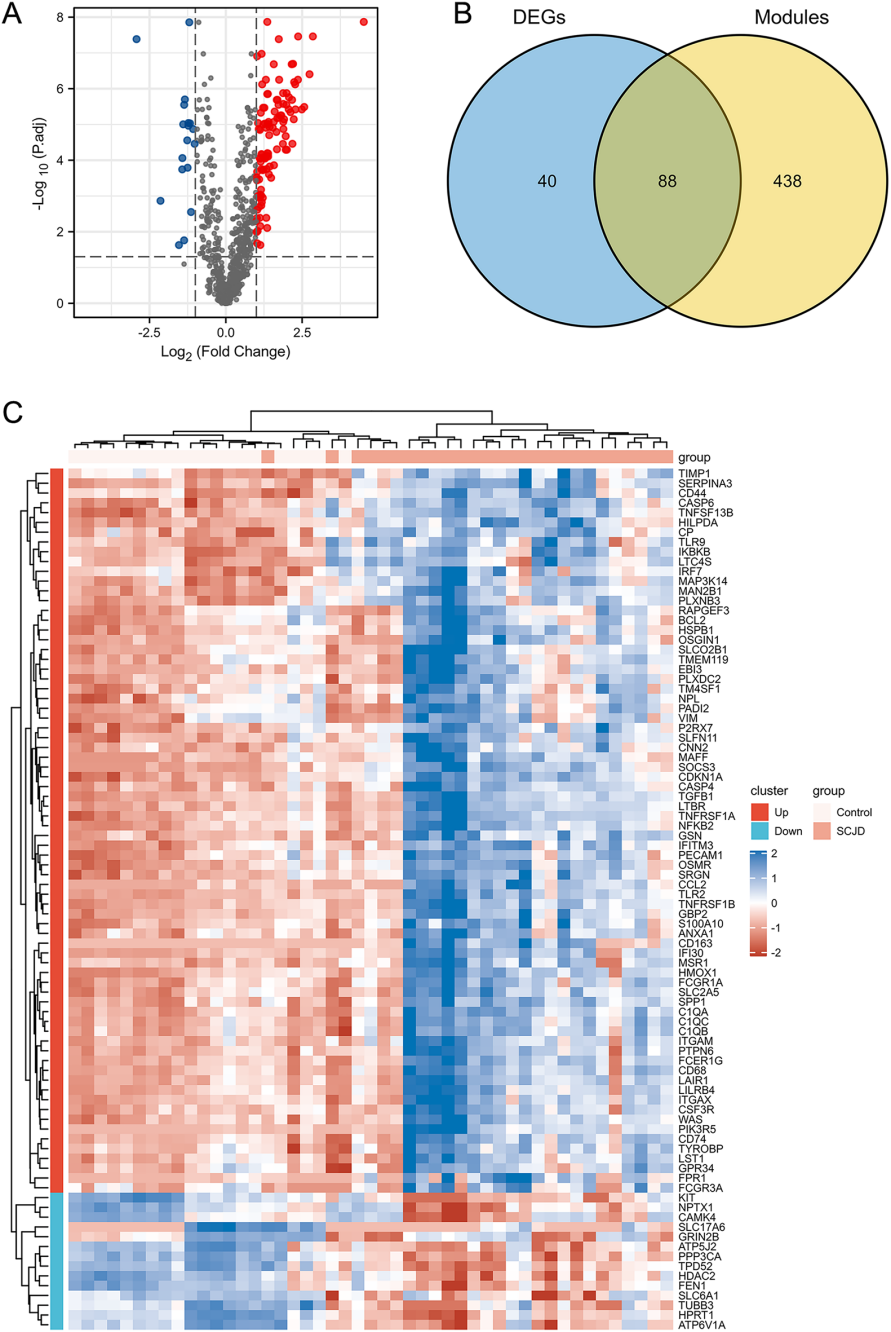
Identification of differentially expressed genes (DEGs) and Key genes associated with neuroinflammation in SCJD. (**A**) Volcano plot of DEGs between control and SCJD samples. (**B**) The Venn plot of 88 genes shared between the key module genes and DEGs. (**C**) The expression levels of 88 common genes in neuroinflammation from SCJD.

After differential analysis using the “limma” package, 128 DEGs of SCJD were obtained, including 110 upregulated DEGs and 18 downregulated DEGs, as shown in Fig. 3A. Red represents upregulated DEGs, blue represents downregulated DEGs, and gray represents non-DEGs. The Venn diagram package identified 88 genes shared between the key module genes and DEGs in SCJD (Fig. 3B). The expression levels of these 88 genes were visualized using the “Complex Heatmap” package (Fig. 3C).

### Functional enrichment analysis

**Figure 4:**
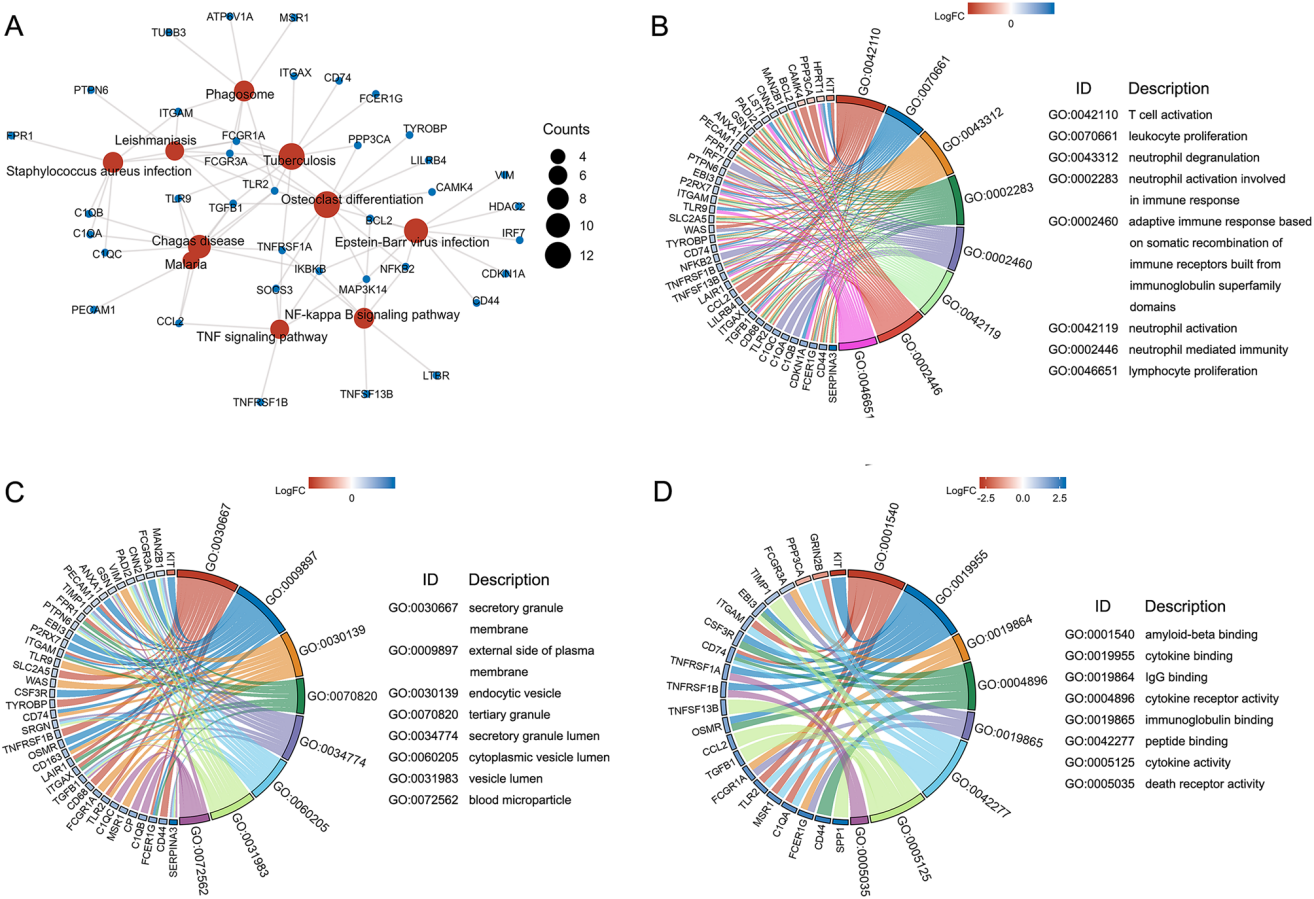
Functional enrichment analysis of 88 genes in neuroinflammation from SCJD. (**A**) Top 10 KEGG pathway enrichment results of 88 genes. (**B**) The top 10 pathways in the Biological Process of 88 genes in GO annotation. (**C**) The top 10 pathways in the Cellular Component of 88 genes in GO annotation. (**D**) The top 10 pathways in the Molecular Function of 88 genes in GO annotation. KEGG: Kyoto Encyclopedia of genes and genomes; GO: gene ontology.

The KEGG pathway analysis results indicate that the 88 candidate genes are mainly enriched in biological signaling pathways such as Osteoclast differentiation, Tuberculosis, Chagas disease, Epstein-Barr virus infection, Staphylococcus aureus infection, NF-kappa B signaling pathway, Leishmaniasis, Malaria, Phagosome, and TNF signaling pathway (www.kegg.jp/kegg/kegg1.html) (Fig. 4A). In addition, the GO analysis of the 88 candidate genes revealed that these genes are mainly enriched in biological functions related to the activation of immune cells and the binding of immune proteins, including T cell activation, leukocyte proliferation, neutrophil degranulation, neutrophil activation involved in immune response, neutrophil activation, neutrophil mediated immunity, lymphocyte proliferation, mononuclear cell proliferation, and response to interferon-gamma, etc., as shown in Fig. 4B. The CC analysis shows that the gene products are predominantly located in places such as secretory granule membrane, external side of plasma membrane, endocytic vesicle, tertiary granule, secretory granule lumen, cytoplasmic vesicle lumen, vesicle lumen, blood microparticle, plasma membrane receptor complex, and platelet alpha granule, as shown in Fig. 4C. In the MF analysis, these genes are mainly enriched in activities such as amyloid-beta binding, cytokine binding, IgG binding, cytokine receptor activity, immunoglobulin binding, peptide binding, cytokine activity, death receptor activity, amide binding, 1-phosphatidylinositol-3-kinase regulator activity, as shown in Fig. 4D.

### Analysis of immune cell infiltrations

**Figure 5:**
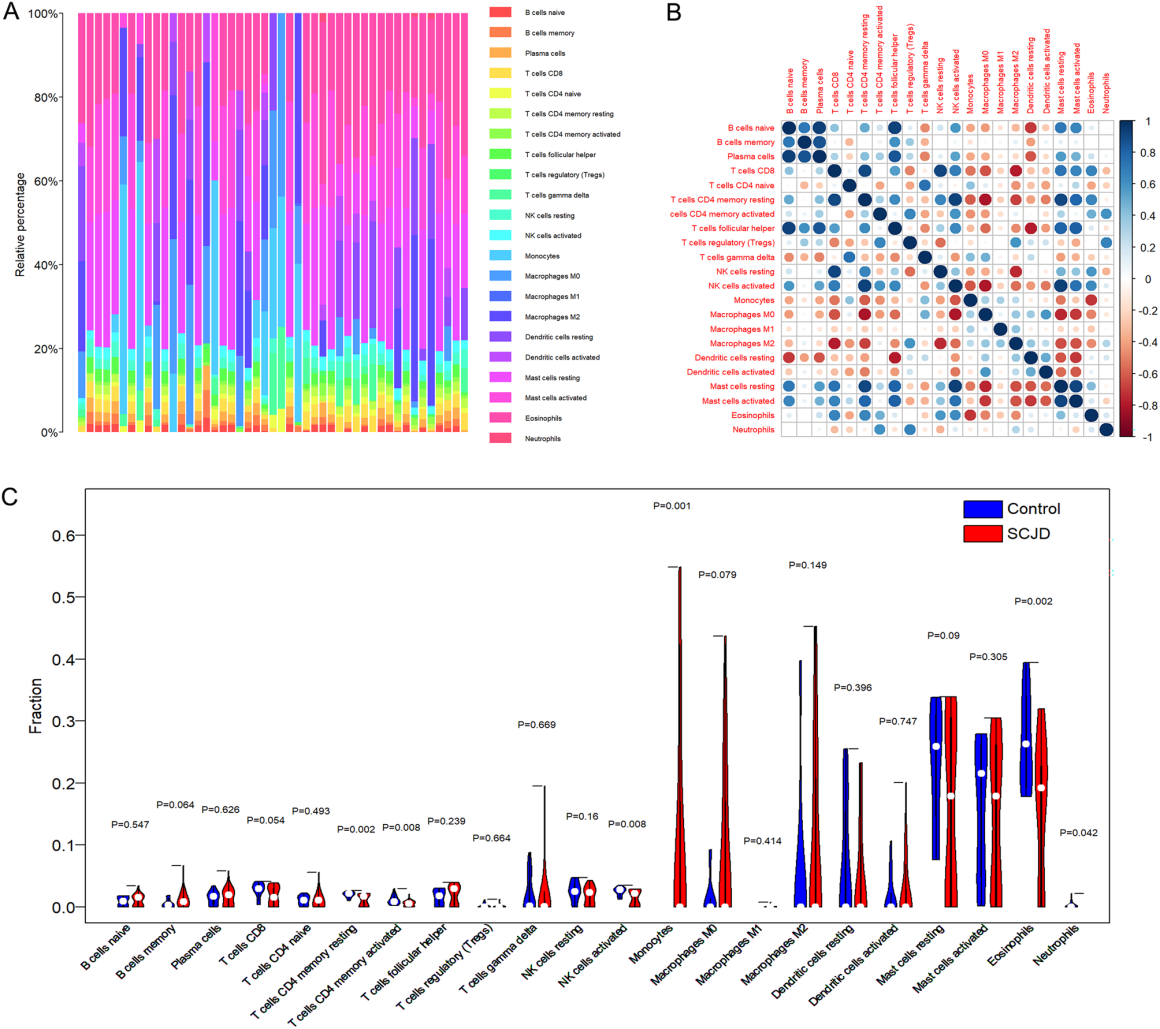
Analysis of immune cell infiltrations of 88 genes in neuroinflammation from SCJD. (**A**) The proportion of immune cells in the tissue. Different colors represent different immune cells. The larger the proportion of the same color in the histogram, the higher the content of the immune cells in the tissue. (**B**) Correlation analysis of immune cells in the SCJD group. (**C**) Violin plot of differences in immune infiltration between SCJD and control groups.

To illustrate whether patients with SCJD had altered immune system activity, we conducted an immune infiltration analysis and found significant differences in the abundances of 22 immune cell subtypes (Fig. 5A). Among them, correlation analysis results suggested that T cells CD4 memory resting has a strong positive correlation with T cells CD8 and NK cells activated and has a strong negative correlation with Macrophages M0, NK cells activated has a strong positive correlation with Mast cells resting and has a strong negative correlation with Macrophages M0, Eosinophils has a robust negative correlation with Monocytes (Fig. 5B), revealing that immune cells activation might be the crucial pathological mechanism causing SCJD progression. Additionally, the infiltration levels of T cells CD4 memory resting, T cells CD4 memory activated, NK cells activated, and eosinophils were markedly lower in SCJD patients. In contrast, the infiltration levels of Monocytes and Neutrophils were appreciably higher in SCJD patients (Fig. 5C), indicating the altered activity of the immune system might be involved in the onset and progression of SCJD.

### Construction of PPI and screening key genes

**Figure 6:**
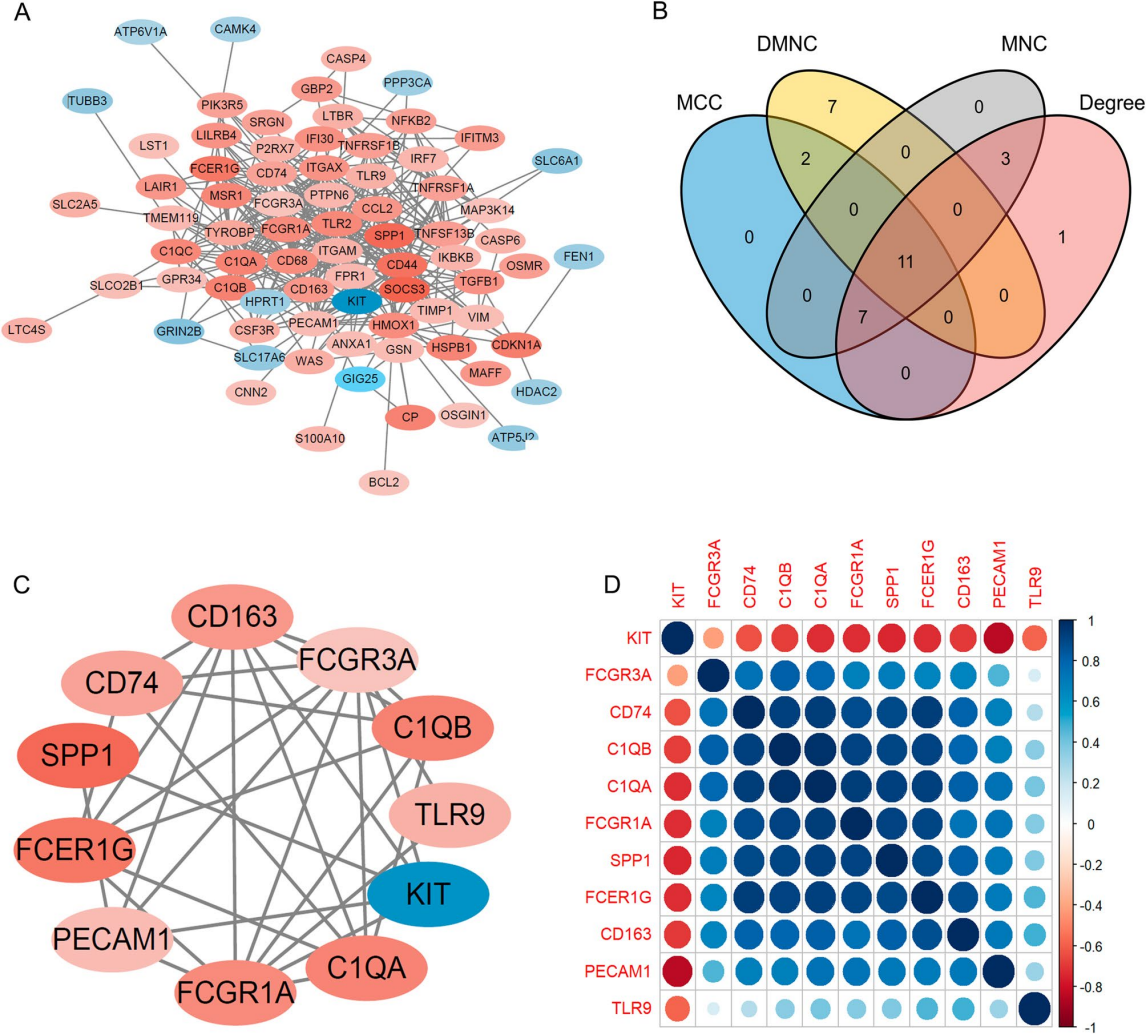
Construction of PPI and screening of key genes. (**A**) Construction of PPI using 88 genes. (**B**) The Venn plot of the intersection between the Top 20 genes of four algorithms (DMNC, MCC, MNC, and Degree). (**C**) The 11 key genes of SCJD in PPI. (**D**) Pearson correlation coefficient among 11 key genes of SCJD. DMNC: density of maximum neighborhood component; MCC: maximal clique centrality; MNC: maximum neighborhood component.

This study introduced 88 candidate genes of SCJD into the online STRING database to construct the PPI network, as shown in Fig. 6A. The network consisted of 88 nodes and 370 edges. The CytoHubba plug-in of Cytoscape software was utilized to identify the hub genes from these 88 candidate genes based on four scoring algorithms: Degree, MNC, MCC, and DMNC, as depicted in Fig. S1. The top 20 genes identified by the four algorithms were selected as the key genes of SCJD (Fig. 6B). Finally, the researchers obtained 11 essential genes (Fig. 6C) and evaluated their co-expression relationships using Pearson correlation coefficient analysis (Fig. 6D).

### Screening KIT (CD117) and SPP1 as hub genes in SCJD

**Figure 7:**
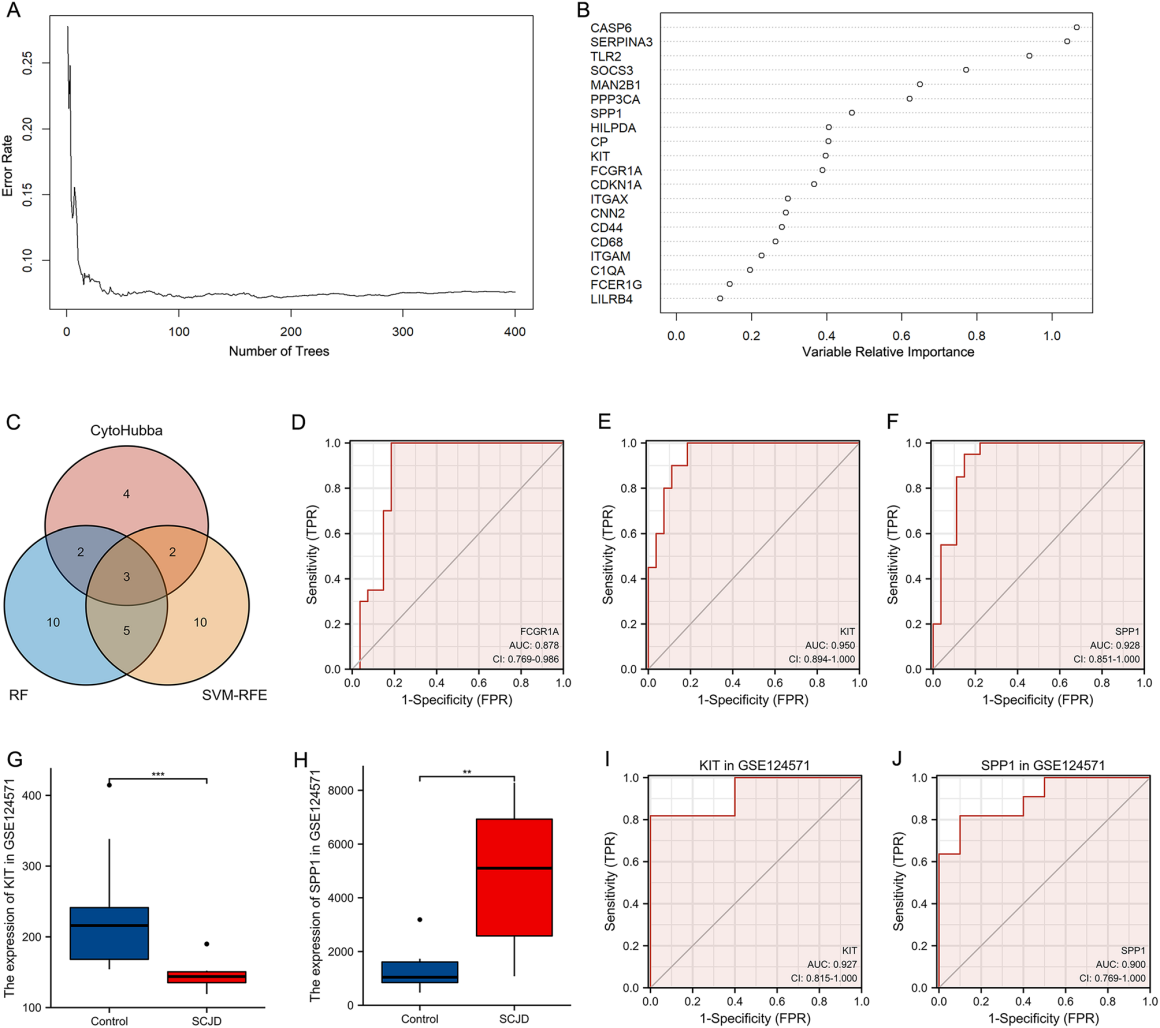
Screening KIT (CD117) and SPP1 as Hub Genes in neuroinflammation from SCJD. (**A**) Random Forest (RF) algorithm. (**B**) Ranking of 20 genes in RF algorithm. (**C**) Venn diagram showing the overlaps in the candidate genes identified using the three algorithms. (**D**) ROC curve validated the sensitivity and specificity of FCGR1A as a predictive biomarker for SCJD. (**E**) ROC curve validated the sensitivity and specificity of KIT as a predictive biomarker for SCJD. (**F**) ROC curve validated the sensitivity and specificity of SPP1 as a predictive biomarker for SCJD. (**G**) The expression of KIT in GSE124571. (**H**) The expression of SPP1 in GSE124571. (**I**) ROC curve validated the sensitivity and specificity of KIT as a predictive biomarker for SCJD in GSE124571. (**J**) ROC curve validated the sensitivity and specificity of SPP1 as a predictive biomarker for SCJD in GSE124571. (*p < 0.05; **p < 0.01; ***p < 0.001; ns: the difference is not statistically significant).

We utilized both the SVM-RFE and RF algorithms to screen further the candidate genes related to SCJD inflammation. After fivefold cross-validation, the SVM-RFE algorithm selected the top 20 feature genes, while the RF algorithm identified the top 20 feature genes. The RF algorithm was used for machine learning to obtain the top 20 feature genes for further analysis (Fig. 7A,B). The top 20 feature genes identified by the SVM-RFE algorithm were also potential markers of SCJD (Supplementary Table 1). Three genes were present in the intersection of the 11 key genes of cytoHubba, the top 20 feature genes of the RF algorithm, and the top 20 feature genes of the SVM-RFE algorithm (Fig. 7C). We evaluated the diagnostic efficacy of these three genes in SCJD using the AUC value. The AUC value of FCGR1A was 0.878 (Fig. 7D), while the AUC value of KIT and SPP1 were above 0.9 (Fig. 7E,F). Finally, KIT (CD117) and SPP1 were found to have higher diagnostic efficiency for SCJD, making them the hub genes of SCJD.

### Expression of KIT (CD117) and SPP1 in SCJD of GSE124571 dataset

KIT (CD117) and SPP1 were validated in the SCJD-related GSE12457 dataset. The results showed that compared with healthy controls, the KIT (CD117) expression was significantly downregulated. In contrast, the expression of SPP1 was significantly upregulated in the brain tissues of SCJD patients, consistent with the expression trend in the combined microarray dataset (Fig. 7G,H). Additionally, the AUC values showed that KIT (CD117) and SPP1 had high diagnostic efficacy for SCJD (AUC > 0.9) (Fig. 7I,J, Table 2).

**Table 2:** The hub genes in Sporadic Creutzfeldt–Jakob disease.

Gene	GeneCards Identifier*	Full name of the Gene	Gene-related Diseases*	Potential roles in SCJD
KIT (CD117)	GC04P054657	KIT Proto-Oncogene, Receptor Tyrosine Kinase	Piebald Trait; Gastrointestinal Stromal Tumor; Mastocytosis, Cutaneous; Testicular Germ Cell Cancer; Testicular Germ Cell Tumor	may lead to the aggravation of neurodegeneration
SPP1	GC04P087975	Secreted Phosphoprotein 1	Pediatric Systemic Lupus Erythematosus; Systemic Lupus Erythematosus	may promote the occurrence of neuroinflammation

*From the GeneCards database (www.genecards.org).

### Exploring the functions of hub genes and molecular docking analysis

**Figure 8:**
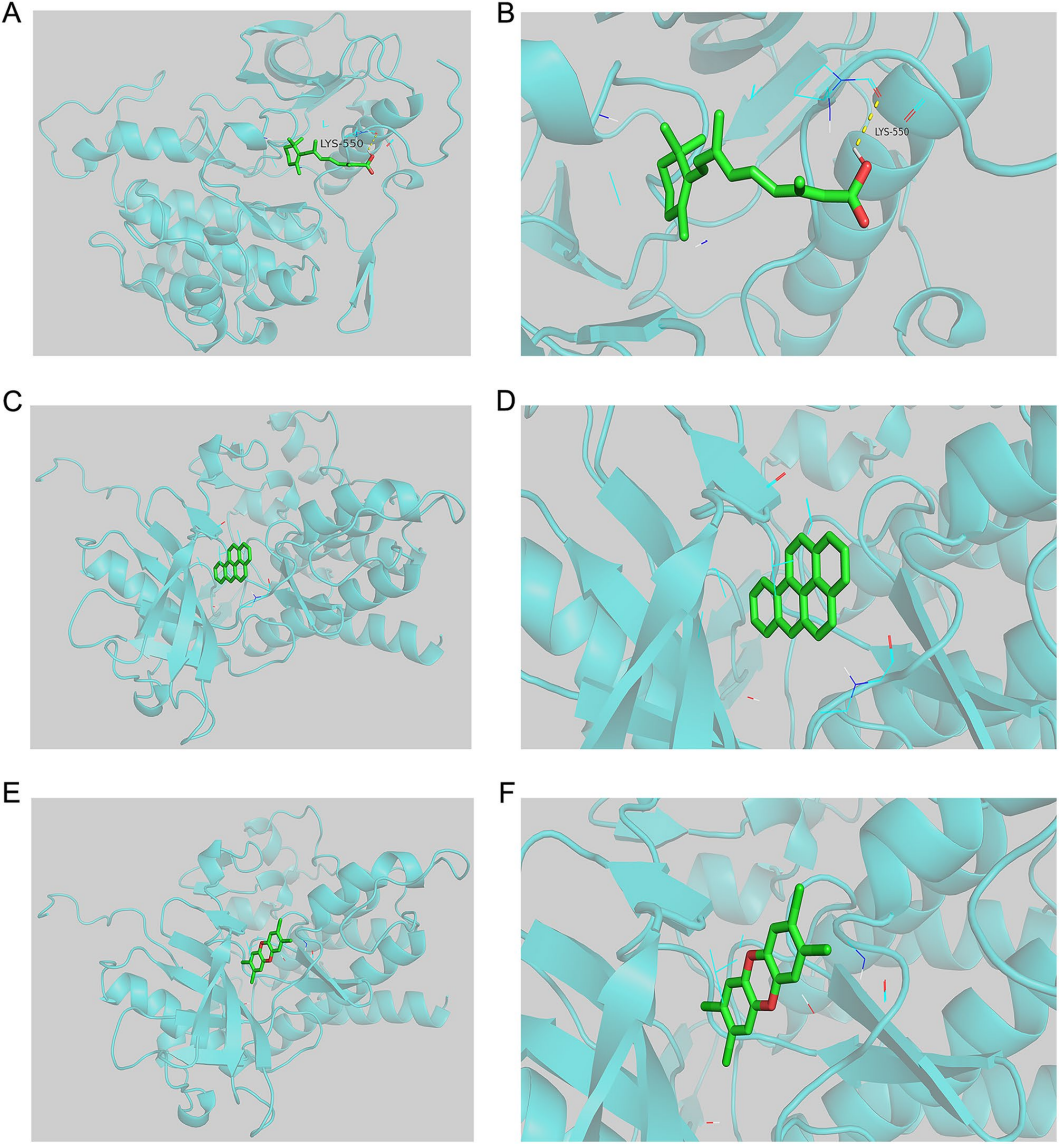
The results of molecular docking between KIT and chemicals (Tretinoin, Tetrachlorodibenzodioxin, and Benzo(a)pyrene). (**A**–**B**) The Molecular docking between KIT and Tretinoin. (**C**–**D**) The Molecular docking between KIT and Tetrachlorodibenzodioxin. (**E**–**F**) The Molecular docking between KIT and Benzo(a)pyrene.

**Figure 9:**
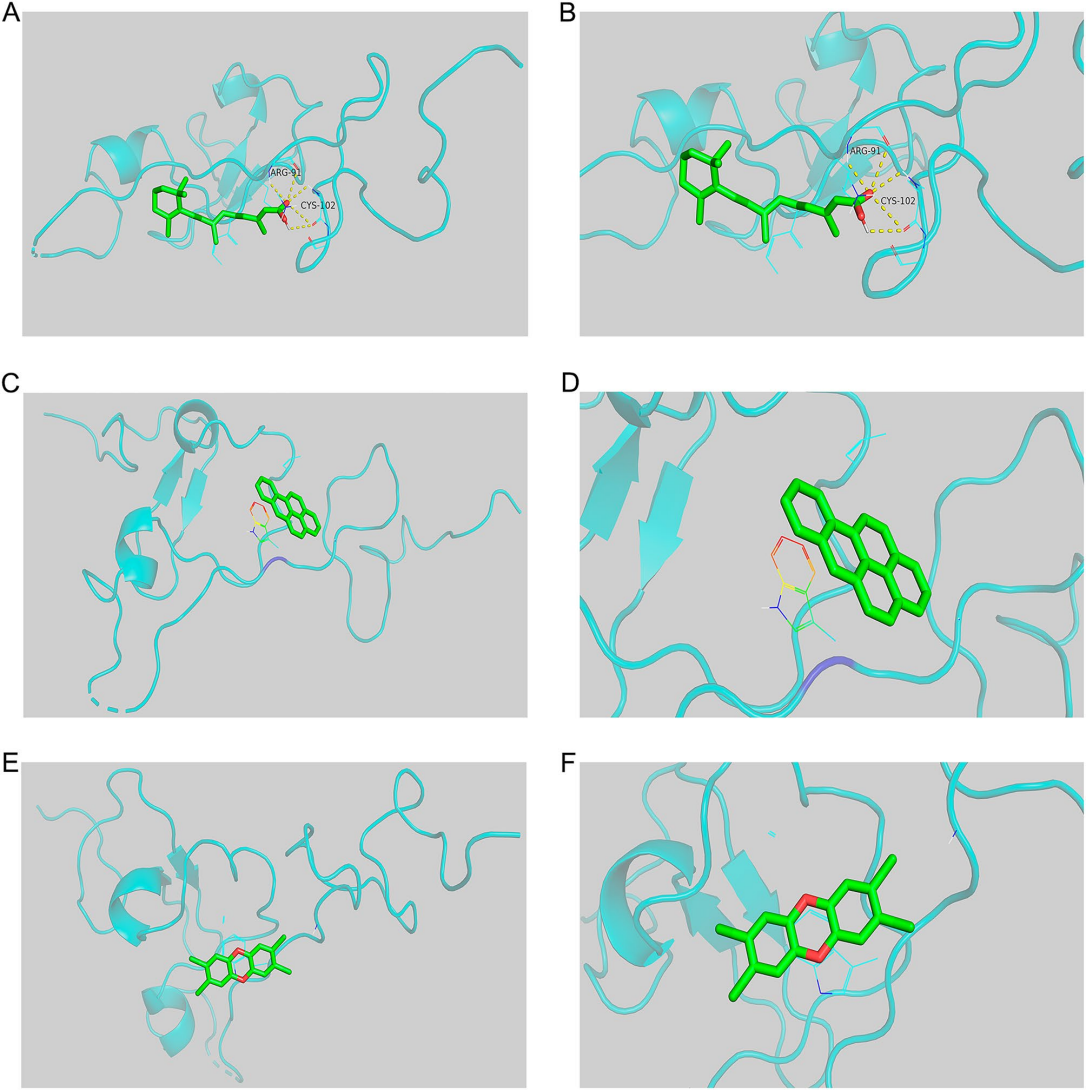
The results of molecular docking between SPP1 and chemicals (Tretinoin, Tetrachlorodibenzodioxin, and Benzo(a)pyrene). (**A**–**B**) The Molecular docking between SPP1 and Tretinoin. (**C**–**D**) The Molecular docking between SPP1 and Tetrachlorodibenzodioxin. (**E**–**F**). The Molecular docking between SPP1 and Benzo(a)pyrene.

To predict the chemicals related to hub genes in CTD, we took the intersection of the top 10 chemicals associated with KIT (CD117) and SPP1. We found that they were both related to Tretinoin, Tetrachlorodibenzodioxin, and Benzo(a)pyrene (Fig. S1E and Supplementary Table 2). Therefore, we analyzed the relationship between hub genes and chemicals using molecular docking methods. We speculate that KIT (CD117) and SPP1 may be the targets of Tretinoin, Tetrachlorodibenzodioxin, and Benzo(a)pyrene in treating SCJD. Therefore, we docked KIT (CD117) and SPP1 with Tretinoin, Tetrachlorodibenzodioxin, and Benzo(a)pyrene to verify our conjecture. The binding energy of ligand-receptor binding conformation is the lowest, indicating that this conformation is the most stable, so the possibility of interaction is the greatest. Figure 8 shows the smaller binding energy of molecular docking result between KIT and Tretinoin (Fig. 8A,B), Tetrachlorodibenzodioxin (Fig. 8C,D), and Benzo(a)pyrene (Fig. 8E,F), revealing that KIT could dock with Tretinoin well (Fig. 8A,B). The smaller binding energy of molecular docking results between SPP1 and Tretinoin (Fig. 9A,B), Tetrachlorodibenzodioxin (Fig. 9C,D), and Benzo(a)pyrene (Fig. 9E,F) were shown in Fig. 9, and the results suggested that SPP1 could dock with Tretinoin well (Fig. 9A,B). Therefore, KIT (CD117), SPP1, and Tretinoin play a vital role in the neuroinflammatory aspect of SCJD, providing new ideas for treating SCJD.

## Discussion

According to related research reports, biological processes such as immune system response, metabolism, developmental biology, and vesicle-mediated transport are involved in the onset of Creutzfeldt–Jakob disease (CJD)^11^. However, there are few reports on the neuroinflammatory mechanisms associated with SCJD pathogenesis, and the specific mechanism is unclear. Therefore, we used a dataset related to SCJD neuroinflammation to explore the mechanism and key genes involved in neuroinflammation during the pathogenesis of SCJD. First, the WGCNA method was used to analyze 800 neuroinflammatory-related genes in SCJD, and 526 genes were obtained from the key modules. Another technique involved using the limma package to conduct DEGS analysis on two groups of samples, resulting in a total of 128 DEGs. We conducted enrichment and immune infiltration analyses on the 88 most central DEGs in the vital module genes and DEGs. The enrichment analysis results suggested that these genes are mainly related to bacterial and viral infections, activation and changes in immune cell states, and other related functions. Immune cell infiltration analysis suggested that immune cell activation and altered activity of the immune system might be involved in the progression of SCJD. Then, after analyzing and verifying the 88 DEGs with various algorithms, KIT (CD117) and SPP1 were determined to be critical genes involved in SCJD neuroinflammation. When exploring the function of key genes using CTD, it was found that KIT (CD117) and SPP1 are related to Tretinoin, Tetrachlorodibenzodioxin, and Benzo(a)pyrene. Finally, molecular docking methods confirmed that KIT (CD117) and SPP1 interact well with Tretinoin. Therefore, KIT (CD117), SPP1, and Tretinoin play crucial roles in the neuroinflammation aspect of SCJD.

Although there have been relatively few studies on SCJD-related neuroinflammation in the past, the role of neuroinflammation in the development of SCJD has been addressed. In some researches on cerebrospinal fluid markers in patients with neurodegenerative dementia, it was found that YKL-40 (also known as Chitinase 3-like 1, a disease-specific marker of neuroinflammation) was significantly increased in SCJD^17^. The expression of sTREM2 (the soluble form of TREM2, TREM2 is an innate immune cell surface receptor that regulates microglial function) was associated with PRNP codon 129 and subtypes, CSF14-3–3 positive, total-tau and YKL-40, and increased with the progression of the disease^18^. In addition, the expression of inflammation-related genes such as chitotriosidase 1 (CHIT1), glial fibrillary acidic protein (GFAP)^19^, and SERPINA1 (α-1 antitrypsin)^20^ in cerebrospinal fluid of patients with SCJD was also increased. Also, there are some studies on the mechanism of SCJD.A study on mice infected with 22L (astrocyte-associated) and ME7 (neuron-associated) PrPSc strains found that PrPSc gene expression characteristics are unrelated to brain region or cell affinity^21^. The latest research on SCJD neuroinflammation found that the impact of SCJD subtypes may not be the strongest or only factor determining the intensity of neuroinflammatory gene expression^7^. Still, specific gene expressions were found to be regulated differently in different brain regions. Before this, some reports suggested that differences in regional gene regulation depend on the patient's genotype at the PRNP codon 129^22,23^. In addition, research has indicated that PrPSc strains and 129 polymorphic codons also affect local microglial activation, as their study on the distribution of activated microglia in different SCJD subtypes showed that microglial proliferation, PrPSc deposition, and spongiform changes were correlated, but not statistically significant^24^. Most importantly, in this study, we used WGCNA to cluster analyze neuroinflammatory genes and SCJD subtypes and found that the strength of neuroinflammatory gene expression was not significantly related to subtypes. Therefore, we believe that standard neuroinflammation-related gene regulations in different brain regions contribute to neuroinflammation in SCJD. Finally, we identified KIT (CD117) and SPP1 as crucial genes in SCJD neuroinflammation.

SPP1 has been reported as a candidate gene for SCJD neuroinflammation^7^. It upregulates the expression of interferon-γ and interleukin-12 while reducing the production of interleukin-10, leading to a type I immune response characterized by solid phagocytic activity^25,26^. SPP1 is a crucial factor that regulates damaged nerve degeneration and regeneration through the c-Fos, PKCα, and p-ERK/ERK pathways^27^. In addition, SPP1 is upregulated in an Alzheimer's disease mouse model and serves as a fundamental regulator of macrophage phenotype and their ability to clear pathogenic β-amyloid protein^28,29^. The high expression of SPP1 inhibits the secretion of IL-10 and may promote the occurrence of neuroinflammation in SCJD. However, its role in SCJD is still uncertain. Another crucial gene is KIT (CD117), a marker for mast cells that plays a vital role in cell proliferation, survival, apoptosis, movement, adhesion, and angiogenesis^30^. Studies have reported that KIT (CD117) expression is reduced in Spinocerebellar ataxia type 3, a neurodegenerative disease, which is consistent with the results of this study^31^. Although there is currently limited research on KIT (CD117) in the nervous system, mast cells have recently been recognized as inducers of neurodegeneration in several diseases^32^. Mast cells are potent activators and regulators of the peripheral immune system and can come into contacting with neuronal tissue. They can affect the blood–brain barrier and may be involved in the pathology of the central nervous system. Mast cells have been associated with major nervous system diseases such as multiple sclerosis, Parkinson's disease, and stroke^33^. The low expression of KIT in SCJD results in the decrease of mast cell markers, which may lead to the aggravation of neurodegeneration. Therefore, the role of KIT (CD117) and mast cells in SCJD will be studied further.

In the process of this study, we found that there are still some candidate genes that are more important, although they are not listed as key genes in this study. It has been reported that prion infection stimulates pattern recognition receptors (PRR)-related molecules and signaling pathways, including Toll-like receptors (TLRs), interferon regulatory factors (IRFs) and some cytokines^34–37^. TLR2 and TLR9 occupy a special position in TLR family and can be used to detect PAMP^38^. It was reported that the mutant TLR4 transgenic mice were inoculated with prions, and it was found that the TLR4 signal pathway had a protective effect on prion infection, and the transcription factor IRF-3 downstream of TLR4 signal showed resistance to prions^39^. These findings suggest that the TLR signal pathway of the innate immune system may regulate prion invasion. Maybe TLR2 and TLR9 play a key role in neuroinflammation of SCJD. In addition, C1qA, C1qB and C1qC are important parts of C1q. C1q is the recognition component of the classical complement activation pathway subunit complex C1^40^. It has been reported that the expression of complement protein is up-regulated in the early stage of neuronal injury, during the aging period and neurodegenerative diseases such as Alzheimer's disease^41^. The synaptic elimination mediated by the overexpression of complement proteins is related to neuronal degeneration^42^ and cognitive loss and schizophrenia in mouse models of various aging and neurological diseases^43–47^. C1qA is not only highly expressed in the midbrain of schizophrenic patients with high inflammation^48^, but also associated with the late onset of familial amyloid polyneuropathy^49^. Therefore, there may be a great correlation between C1qA, C1qB and neuroinflammation of SCJD.

Finally, we found that both KIT (CD117) and SPP1 docked well with Tretinoin, and Tretinoin may be a target for treating SCJD neuroinflammation. Tretinoin (all-trans retinoic acid) is a metabolite of natural vitamin A that can activate three nuclear retinoic acid receptors (retinoic acid receptor α, retinoic acid receptor β, and retinoic acid receptor γ)^50^. These receptors can alter gene expression, protein synthesis, and the growth and differentiation of epithelial cells. Decades of research have established topical retinoic acid as a first-line drug for treating acne vulgaris (AV)^51^. Inducing the expression of RARβ has also been shown to inhibit the carcinogenesis of some squamous cell tumors, including lung cancer, esophageal cancer, and breast cancer^52–57^. However, there are almost no reports on its use in neurological diseases. The idea that Tretinoin may be a target for treating SCJD neuroinflammation still needs more research to be confirmed. Next, we will explore the role of neuroinflammation in the pathogenesis and treatment of SCJD through KIT (CD117), SPP1, and Tretinoin.

### Supplementary Information

Supplementary Information 1.

Supplementary Information 2.

Supplementary Information 3.

Supplementary Information 4.

Supplementary Information 5.

Supplementary Information 6.

Supplementary Information 7.

Supplementary Information 8.

## Data Availability

GSE160208 GSE124571 datasets are from the GEO (Gene Expression Omnibus, https://www.ncbi.nlm.nih.gov/geo/) database.
