# Supplementary material for: Genetic analysis of potential biomarkers and therapeutic targets in neuroinflammation from sporadic Creutzfeldt–Jakob disease
Source: Sci Rep. 2023 Aug 29;13:14122. doi: 10.1038/s41598-023-41066-9 (PMC10465546; doi:10.1038/s41598-023-41066-9)
Supplement: Supplementary file 1 — Supplementary Information 1. [file 41598_2023_41066_MOESM1_ESM.docx]

Supplementary Material

**Genetic Analysis of potential biomarkers and therapeutic targets in Neuroinflammation from Sporadic Creutzfeldt-Jakob Disease**

Yajing Cheng^1^, Ting Chen^2^, and Jun Hu^1^*

1 Department of Neurology, Peking University Shenzhen Hospital, Shenzhen, China

2 Department of Neurology, Shenzhen Second People's Hospital, Shenzhen, China

*Correspondence: Corresponding Author: Jun Hu, Department of Neurology, Peking University Shenzhen Hospital, Shenzhen, China. E-mail: [dochj@163.com](mailto:dochj@163.com)


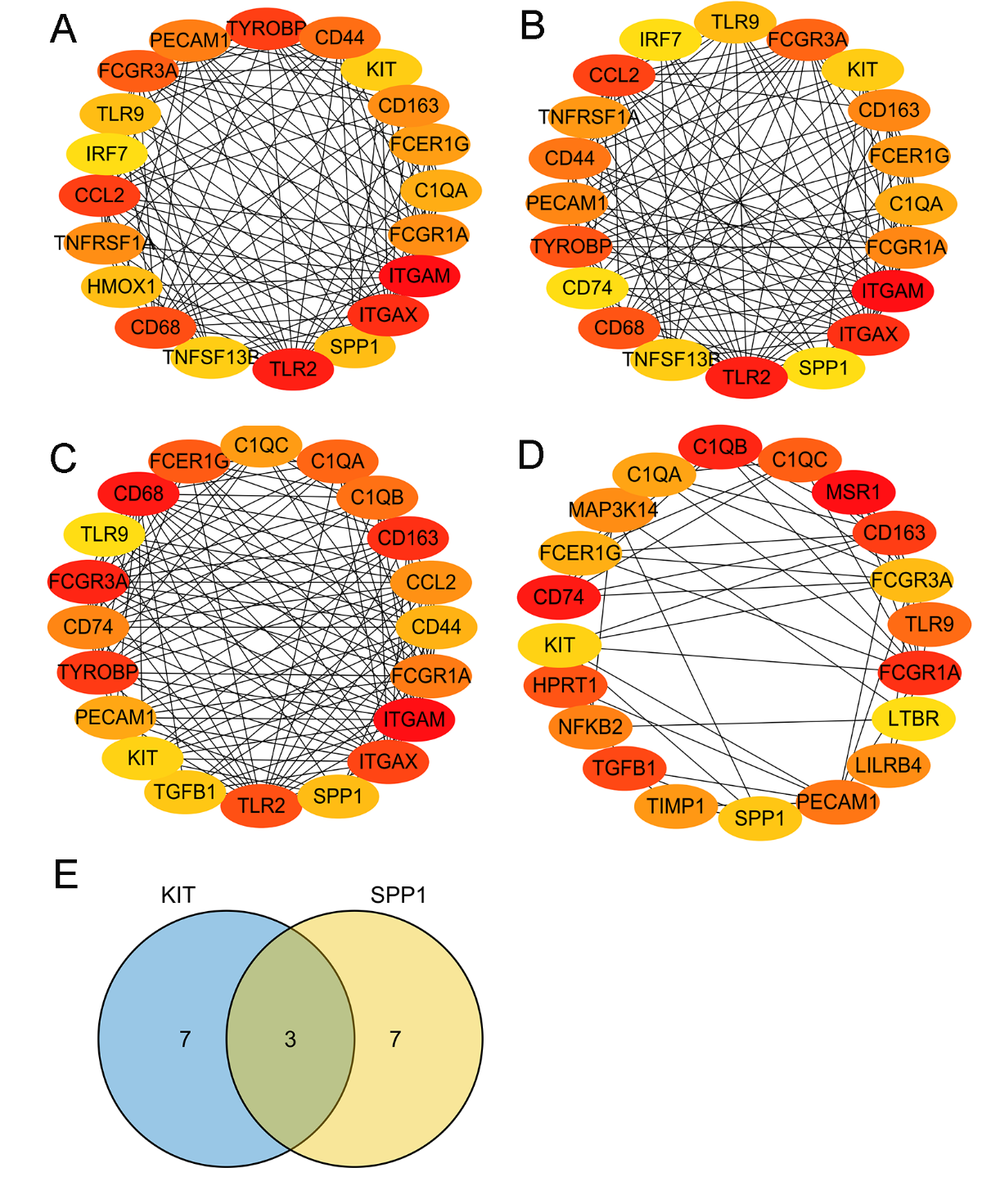


**Figure S1. Construction of PPI and screening of** **the chemicals related to hub genes.** A. The Top 20 genes of Degree algorithm in PPI. B. The Top 20 genes of the MNC algorithm in PPI. C. The Top 20 genes of the MCC algorithm in PPI. D. The Top 20 genes of the DMNC algorithm in PPI. E. The Venn plot of the intersection of the chemicals is all related to hub genes. MNC: maximum neighborhood component; DMNC: density of maximum neighborhood component; MCC: maximal clique centrality.

**Supplementary Table1.** The SVM-RFE algorithm identified the top 20 feature genes.

| Feature Name | **Feature ID** | **Avg. Rank** |
| --- | --- | --- |
| **SPP1** | 11 | 6.3 |
| TUBB3 | 46 | 8.3 |
| LILRB4 | 56 | 9.2 |
| TPD52 | 26 | 12.3 |
| CD68 | 29 | 13.3 |
| KIT | 6 | 13.6 |
| FCGR1A | 32 | 14 |
| SLC6A1 | 65 | 15.4 |
| FCGR3A | 86 | 15.4 |
| OSMR | 12 | 17 |
| PPP3CA | 3 | 18.2 |
| C1QC | 24 | 18.2 |
| SERPINA3 | 2 | 18.8 |
| SOCS3 | 4 | 19.4 |
| LST1 | 73 | 20.1 |
| PADI2 | 84 | 21.5 |
| TLR9 | 39 | 21.8 |
| BCL2 | 83 | 22.1 |
| CSF3R | 68 | 22.7 |
| WAS | 48 | 24 |

**Supplementary Table2.** The Top 10 chemicals related to hub genes in CTD.

| KIT | **SPP1** |
| --- | --- |
| Valproic Acid | Asbestos, Serpentine |
| Arsenic Trioxide | Tetradecanoylphorbol Acetate |
| bisphenol A | Glucose |
| Tretinoin | Nanotubes, Carbon |
| Imatinib Mesylate | Tetrachlorodibenzodioxin |
| Tetrachlorodibenzodioxin | Calcitriol |
| Benzo(a)pyrene | Tretinoin |
| Cisplatin | Ethanol |
| Estradiol | Gentamicins |
| Mechlorethamine | Benzo(a)pyrene |
